# Digital Twin-Based Integrated Monitoring System: Korean Application Cases

**DOI:** 10.3390/s22145450

**Published:** 2022-07-21

**Authors:** Sangsu Choi, Jungyub Woo, Jun Kim, Ju Yeon Lee

**Affiliations:** 1 R&D Research Center, IGI Korea, Seoul 08376, Korea; sangsu.choi@ontwins.com (S.C.); jungyub.woo@ontwins.com (J.W.); 2 IT Converged Process R&D Group, Korea Institute of Industrial Technology, Ansan-si 15588, Korea; jun.kim@kitech.re.kr; 3 Department of Mechanical System Design Engineering, Seoul National University of Science and Technology, Seoul 01811, Korea

**Keywords:** digital twin, cyber-physical systems, smart manufacturing, smart factory, monitoring system

## Abstract

A digital twin is a virtual model of a process, product, or service, which is one of the key technologies in the fourth industry. The pairing of the virtual and physical world allows analysis of data and monitoring of systems to head off problems before they occur. This paper presents a digital twin architecture and a system based on an interoperable data model. It explains how to build a digital twin for the integrated control monitoring using edge devices, data analytics, and realistic 3D visualization. The system allows continuous collaboration between field engineers for data gathering, designers for modeling 3D models, and layout engineers for layout changing by generating 3D digital twin models automatically. The system helps stakeholders focus on their respective roles to build digital twins. Examples applied to the Korean automotive parts makers are also introduced in this paper. The system can be easily used by small and medium-sized enterprises (SMEs) as well as large companies. Beyond simply watching the production site with CCTV, the production site can be intuitively managed based on the digital twin.

## 1. Introduction

With the advent of the fourth industrial revolution, smart factories are built based on cutting-edge information and communication technologies (ICT) such as cloud technology, data analytics using artificial intelligence (AI), internet of things (IoT), and additive manufacturing [1]. Cyber-physical systems (CPS), the core element of a smart factory, is based on end-to-end integration using ICT from procurement, production, marketing, distribution logistics, to services [2,3]. Digital twin is a technology that can predict results by simulating situations with computers. [4]. When digital twins are applied to manufacturing companies, it is reported that process operation costs are reduced by 10–15% and machine maintenance costs are reduced by 15–20% [5]. In addition, it is predicted that digital twins will be applied at a rate of 50% or more when projects are undertaken by large companies in 2021 [5].

It is practically impossible to build an existing factory into an ideal digital twin within a short period of time. It is necessary to build a digital twin according to the real situation step by step. In order to build a digital twin, it is necessary to establish an IT infrastructure that enables real-time data collection in the manufacturing environment. Three-dimensional digital models identical to the field are developed. This digital model and field data are connected so that the current status is monitored in real time. Analytical technologies such as AI and simulation are applied to predict the future. The analyzed results are reviewed by various stakeholders. Decision making is applied in the field. While large corporations announce many best practices for digital twins, small and medium sized enterprises (SMEs) have technical, budget, and human resource hurdles. SMEs are also required to develop a methodology and system to build a digital twin. 

This paper explains a digital twin architecture, a developed system based on an interoperable data model, and case studies of building an integrated monitoring system based on digital twins using edge devices, data analysis, and realistic 3D visualization. The structure of this paper is as follows. Section 2 examines preliminary studies on digital twins and the technical requirements required to build digital twins SMEs. Section 3 describes a digital twin architecture, data model, and developed system functions. Section 4 introduces the implementation cases of a Korean auto parts company, and Section 5 discusses the conclusions and future research.

## 2. Related Research

Research on digital twins is being actively conducted worldwide. Lee et al. [6] suggested a 5C architecture related to digital twins. 5C stands for Connection, Conversion, Cyber, Cognition, and Configure. It is an architecture that supports real-time analysis and quick decision making by connecting the same virtual machine with the physical machine to which several sensors are attached. Alam et al. [7] suggested a cloud based digital twin architecture reference model. Redelinghuys et al. [8] presented a six-layer-based digital twin architecture consisting of local data, IoT gateway, cloud-based database, emulation, and simulation. For digital twin, research on architecture and framework is extremely important because heterogeneous technologies and systems must be connected and integrated. However, there are only a few comprehensive application examples described in architecture and framework. Currently, application cases focusing on specific tasks or technologies are being announced. Zeng et al. [9] pointed out that the digital twin technology is still at a theoretical stage, the application framework and application method are not clear, and the lack of reference application examples is also a problem. They proposed an application framework for digital twins and introduced an example for real-time monitoring of welding production lines. Zhou et al. [10] introduced a framework for intelligent manufacturing, the knowledge-driven digital twin manufacturing cell (KDTMC). Three application cases for intelligent process planning, intelligent production scheduling and production process analysis, and dynamic regulation were also introduced. Choi et al. [11] introduced an application case of an electric parts company with explanation on the digital twin application framework composed of heterogeneous systems and technologies such as manufacturing execution system (MES), product lifecycle management (PLM), process mining (PM), discrete event simulation (DES), and virtual reality (VR). Choi et al. [2] introduced a case of developing an overall equipment efficiency (OEE) dashboard using a reference model developed by the National Institute of Standards and Technology (NIST) and a commercial IoT platform for smart factory establishment. For connection with the worksite, standards application cases such as OPC-UA and MTConnect were introduced [12], and cases laying the foundation for a smart manufacturing environment based on MES were presented [13]. 

Studies on analyzing collected real-time big data by machine learning and deep learning are being actively published. These analyses are mostly applied intensively to the maintenance work of equipment [14]. Simulation is mainly used for process, manufacturing line, and factory analysis. Bottani et al. [15] introduced a case of digital twin connected to physical automated guided vehicles (AGVs) based on a DES engine. Gyulai et al. [16] introduced a digital twin that evaluates various modular cell configurations through DES and applied automated model building and centralized simulation model control. Zhang et al. [17] explained that the introduction of a digital twin enables additional fusion between the physical and virtual spaces in the workplace, enabling dynamic scheduling. In addition, a five-dimensional digital twin consisting of physical entity, evolving virtual entity, digital twin data, on-demand services, and connections for machines in the workplace was introduced. After exploring the digital twin-based machine availability prediction, disturbance detection, and performance evaluation method, a digital twin-enhanced dynamic scheduling methodology was proposed. Research on key indicators for smart manufacturing are also suggested [18], and studies on how to visualize those indicators are also being conducted [19]. Using 3D enables the ability to intuitively grasp the status and has the effect of greatly improving communication with the people who cooperate [11]. Due to these advantages, research and studies are being introduced to visualize factories based on VR/AR using Unity [20]. Typically, VR is mainly used for evaluation of newly constructed factories or changed manufacturing lines, and cases where AR is applied for maintenance of existing factories and equipment are reported [21]. 

Digital twins are being applied in various field. Conejos et al. [22] emphasized that developing a digital twin is a difficult task and requires a continuous adjustment and learning process. They presented an example of a digital twin implementation of a water distribution network in Valencia (Spain) and its metropolitan area (1.6 million citizens). Bonilla et al. [23] introduced a digital twin-based water distribution system and applied and verified the system in two regions of Colombia. Ramos et al. [24] demonstrated to Water Distribution Networks that water savings of up to 28% can be achieved through fast detection of leaks based on a digital twin system. Alves et al. [25] presented a case of digital twin implementation in the agricultural sector. They elaborated a dashboard that enables real-time monitoring and data collection for a soil probe. Angin et al. [26] proposed a low-cost farmland digital twin framework for smart agriculture. The system runs a network of wireless sensors and computer vision algorithms to detect plant diseases, weed colonies, and phytonutrient deficiencies. Bado et al. [27] described an application case of digital twin technology in the field of civil engineering and structural health monitoring (SHM) to extend the lifespan of facility assets. Junquera et al. [28] applied a digital twin-based simulation technology to the rolls replacement process in the steel industry and drew positive results for the automation of the work. A case where the digital twin was applied to the food plant was also introduced. Tancredi et al. [29] described an approach to implement machine learning algorithms in a digital twin environment and apply them to a food plant. They emphasized that digital twin technology is effective for predicting anomalies and enhancing worker safety. Gallala et al. [30] proposed a digital twin approach for human-robot interaction (HRI) based on Industry 4.0-enabled technologies such as mixed reality, Internet of Things, collaborative robots, and artificial intelligence. They validated the proposed method using Microsoft Hololens 2 and the KUKA IIWA collaborative robot, claiming that even people without programming knowledge can achieve human–robot interaction. Digital twin technology is being actively applied in the medical field as well. Digital twin technology was applied for remote surgery, and ideas for establishing communications and necessary cybersecurity technologies were proposed to help develop digital twin architectures [31]. 

The studies and cases mentioned above are methods that can be implemented only in institutes with internal experts or are examples of high-level large companies. Although it is possible to build a digital twin based on solutions from global vendors, most SMEs are experiencing difficulties in implementation due to the cost burden. Furthermore, professional technical experts for simulation, AI, etc., are required, and experts who can directly build a 3D virtual factory using Unity are required. Currently, most SMEs can consider introducing a digital twin-based control monitoring system to intuitively grasp the shop floor, and to do so, the following three technical criteria must be satisfied.

1. Engineering tasks such as factory layout change should be performed by the manufacturing company’s own personnel so that it can quickly reflect the situation of a physical factory that changes frequently in a situation where internal experts are scarce.
2. Even without 3D expertise, 3D models of changing factories should be automatically or conveniently created. Three-dimensional visualization should be supported, all cloud, web, and mobile and should be lightweight so that the entire factory can be rendered. (This is because it takes a lot of costs and integration tasks to implement system functions in each cloud, web, and mobile environment.)
3. Connected to the actual factory site, 3D models, KPIs, and data must be visualized in real time, and various user-friendly functions must be provided for users.

The next section introduces the digital twin-based integrated monitoring system developed to satisfy these three criteria.

## 3. Digital Twin-Based Integrated Monitoring System

As mentioned above, the digital twin is built and utilized in: (1) digital model building, (2) real-time field data connection, (3) monitoring and analysis, (4) stakeholder decision making, and (5) field application procedure. Most SMEs have domain knowledge, but they are weak in the IT technologies area related to the 4th industry [1]. In this study, in order to increase the understanding of digital twin building of SMEs, the tasks and technologies of each layer are separately explained based on a digital twin architecture. In addition, by developing a digital twin solution for SMEs, the technology and tasks of each layer were integrated. 

### 3.1. System Architecture

**Figure 1:**
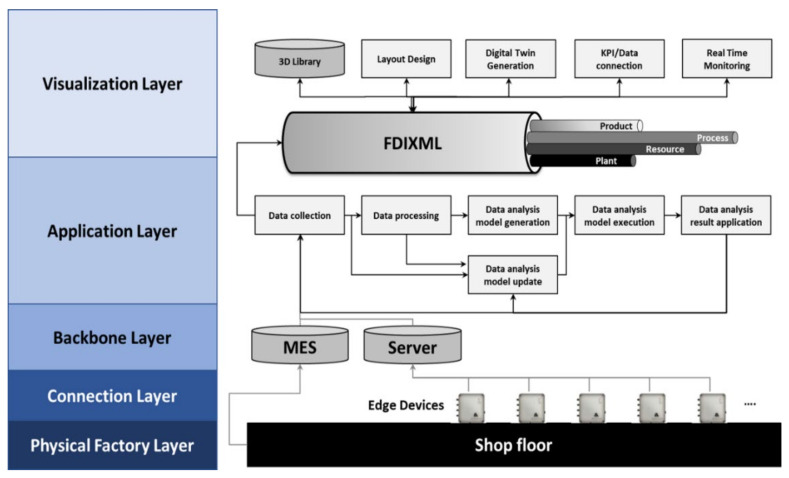
Overall architecture.

As shown in Figure 1, the system is composed of five layers. The physical factory layer is a physical area that consists of machines, material handling systems, and labors including sensors and actuators. The connector layer is an area where edge devices are distributed, and data are collected from the physical factory layer. This layer also includes data collection based on standards such as OPC-UA and MTConnect. The backbone layer is where the MES or IoT platform is located and is responsible for storing and managing big data. The application layer is a part that performs analysis based on big data where analysis applications such as machine learning, deep learning, and simulation are located. The visualization layer includes all data connections with the MES or IoT platform, authoring tools for factory modeling, web/mobile visualization, and VR/AR functions, and an integrated data model for this is located in this layer. The physical factory layer, the connection layer, and the backbone layer are areas where physical elements and systems are set up to make help from experts inevitable. However, the application and visualization layer should be built easily and conveniently so that even users of SMEs can use it. For this, the system must be developed easily and user-friendly based on an integrated data model.

### 3.2. Data Model

**Figure 2:**
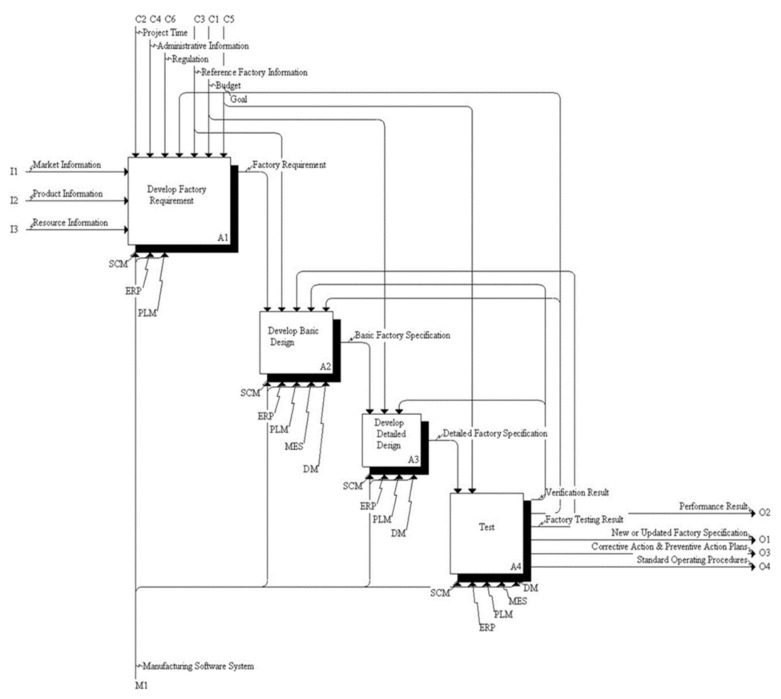
Factory design and improvement (FDI) reference activity model.

As mentioned earlier, the integrated monitoring system must simultaneously support various environments such as cloud, web, and mobile, acquired data from heterogeneous legacy systems from time to time, and support interfaces with various software such as analysis tools. International standards exist for each domain area [32] (e.g., MES, PLM, VR, etc.), but they are not commonly used in the real field except STEP. For flexible integration, it is necessary to develop a data model to build digital twins connecting heterogeneous systems. The data model introduced in this paper was defined based on the FDI reference model developed by the NIST to improve the smart factory design. The FDI reference model is based on the business process related to the factory/manufacturing line/process/machine of a global manufacturing company and is composed of 4 activities and 28 tasks as shown in Figure 2 and is modelled as IDEF0 [33].

**Figure 3:**
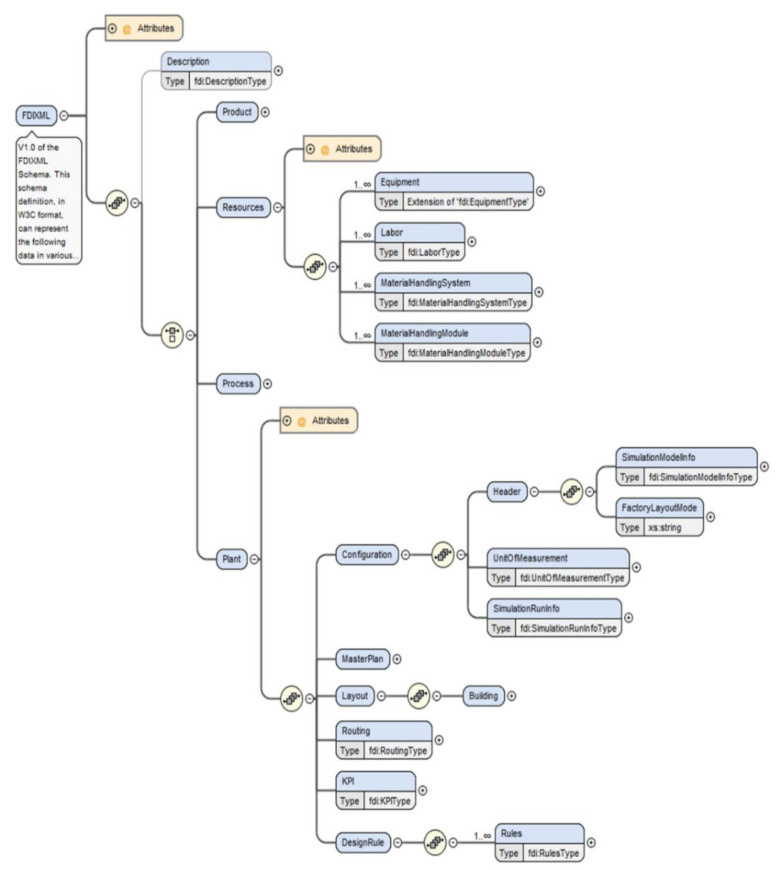
FDI data model schema.

By analyzing the input and output of these activities and tasks, and XML data schema was designed as shown in Figure 3 [34]. Table 1 summarizes the physical elements constituting the factory such as machines, material handling systems, and labors, and logical elements such as process areas, routing, and rules. Each element has its own data type. 

As shown in Figure 3, this schema is a final form in which P3R (Product, Process, Resource, and Plant) data are logically organized based on plant information. Processes are arranged on the layout of the factory, and machines, labors, material handling systems, and material handling modules are configured within or between processes. Through the routing of each process, the final product is produced.

### 3.3. System Functions

#### 3.3.1. Connection and Backbone Layer

The manufacturing company controls the work site based on the MES system. According to MESA [35], MES consists of 11 functions: Resource Allocation Status, Operation/Detail Scheduling, Dispatching Production Units, Document Control, Data Collection/Acquisition, Labor Management, Quality Management, Process Control, Maintenance Management, Product Tracking and Genealogy, and Performance Analysis. These functions are applied in combination according to the size and environment of the enterprise. Despite the establishment of the MES, issues related to data collection and processing at the manufacturing site often arise. Recently, edge devices are being used for passive data collection and efficient processing of large data transmission. By establishing a wireless network-based data transmission environment, it is possible to automatically collect real-time data such as cycle time, production quantity, temperature, humidity, and vibration of the factory. Data collected from edge devices is stored and managed on its own server.

#### 3.3.2. Application Layer

A data analysis module is equipped in the application layer. A typical data analysis process consists of data collection, data preprocessing, data analysis model creation, data analysis model execution, and data analysis model update. In this study, as shown in the architecture of Figure 1, a closed-loop data analysis process flow was made possible [36]. When a data analysis model is created through data collection, the data analysis model can be executed in the data preprocessing stage. The performance of the data analysis model may be degraded by including time series trends or by aspects of the manufacturing system environment such as the life expectance of the machine. Standards for systematic measurement already have been set, enabling continuous data analysis model updates. 

By monitoring the execution results of the data analysis model and the realized output, the field error rate of the data analysis model can be calculated. When it is determined to update the data analysis model, the training data set for generating the data analysis model is reconstructed with recent or changed data, and the model is updated. As the updated data analysis model does not guarantee better performance than the original model, comparison between the newly updated data analysis model and the original model should be conducted first. This comparison determines whether to adopt the updated model or keep the original. When the model is updated, data suitable for the updated model are collected, and the new model is executed. The generated data analysis model supports predictive model mark-up language (PMML), an XML-based predictive model exchange format.

#### 3.3.3. Visualization Layer

A user-friendly visualization system for the digital twin has been developed based on the FDIXML data model. Previously, tasks such as creating a 3D factory based on a 2D layout, establishing an interface between the 3D factory and edge devices/MES, and continuously reflecting the changing factory situation to the digital twin are extremely difficult and expensive. We built a 3D library of resources such as machines, material handling systems, and labors in the factory allowing any user, for easy and quick deployment of the libraries, to automatically create a digital twin. The information that needs to be connected to the actual factory can be intuitively defined by the user on the digital twin, and RESTful API can be automatically generated.

**Figure 4:**
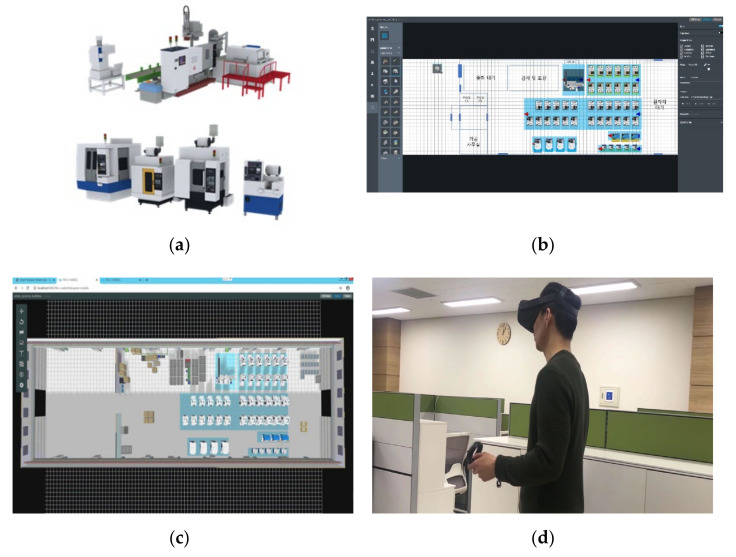

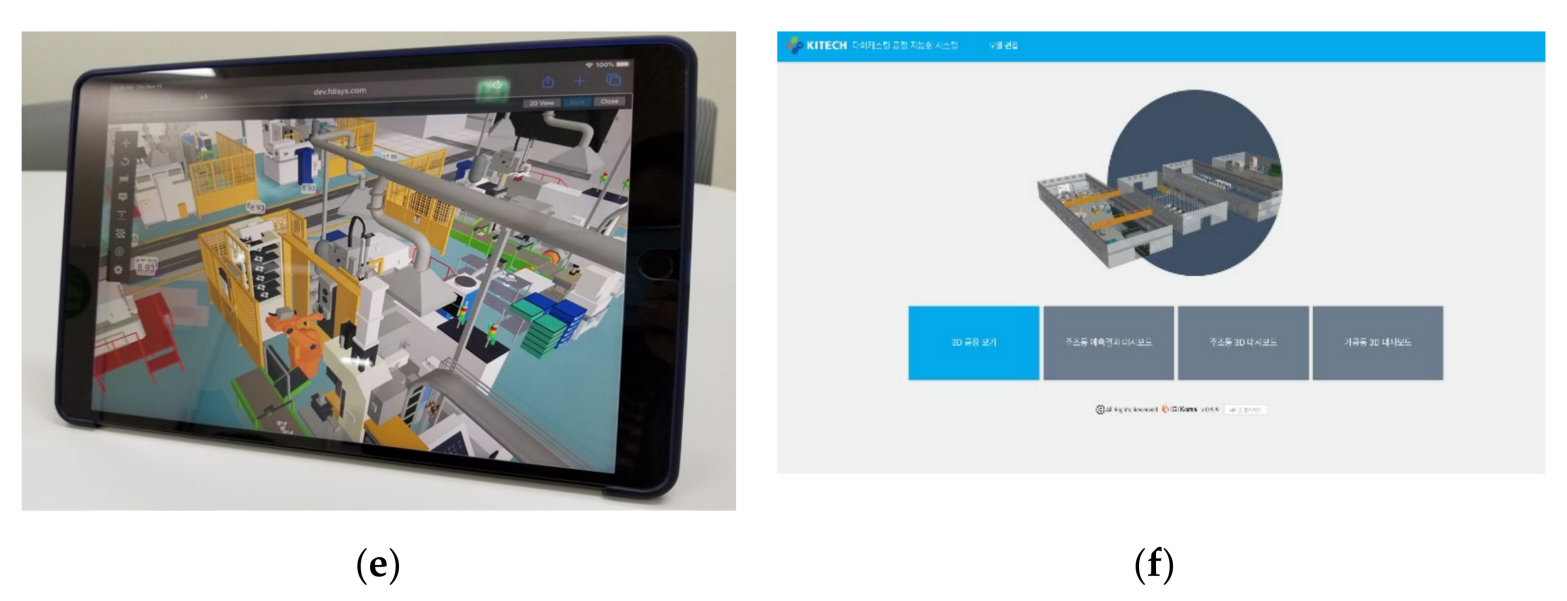
System Functions: (**a**) Resource Library, (**b**) Factory Layout Design Tool, (**c**) Web-based 3D Visualization, (**d**) Design Review using VR, (**e**) Mobile Visualization, (**f**) Dashboard Screen.

As shown in Figure 4b, the user can arrange the factory layout as if playing a game in the process layout authoring tool by using the machines and material handling systems library in the factory shown in Figure 4b. Based on the deployed layout, a 3D virtual factory model is automatically generated, and the realistic 3D factory visualized on the web can be checked through the visualization function as shown in Figure 4c. As pictured in Figure 4d, the factory can be evaluated using VR devices such as Oculus, and the factory can also be evaluated via mobile as shown in Figure 4e. When sensor data and KPIs that need to be connected to the physical factory are defined in the form of types and items, RESTful APIs are automatically created for shop floor connection through MES or IoT platforms. Connected real-time data and KPIs can be checked in real time through the digital twin. Figure 4f shows the screen of the integrated monitoring system implemented. 

When the worksite layout is changed, the user of the manufacturing company can easily change the layout of the virtual factory using the developed authoring tool. Even anyone who can only draw shapes in PowerPoint can change the factory layout. After that, the 3D model is automatically created and the linked KPIs are updated. If the KPIs or data need to be changed, the user can conveniently create/change/delete RESTful APIs. The biggest advantage of this system is that not only experts but also novices can easily use it. Only when new machines or material handling systems are introduced, is it required to build a library with the help of experts on a one-off basis. If SMEs can perform 3D modelling through training and education, they can also build the library on their own. 

## 4. Korean Application Cases

The company “N” is a “metal platform” based provider of metal parts including die-casting, precision machining, surface treatment and assembly. It is a global manufacturing company that has the headquarters in South Korea and operated 12 subsidiaries and factories in Korea and abroad.

**Figure 5:**
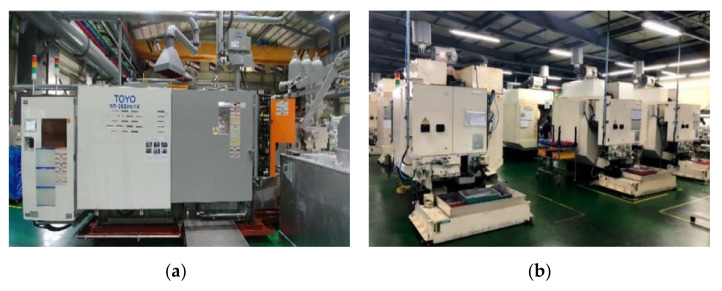

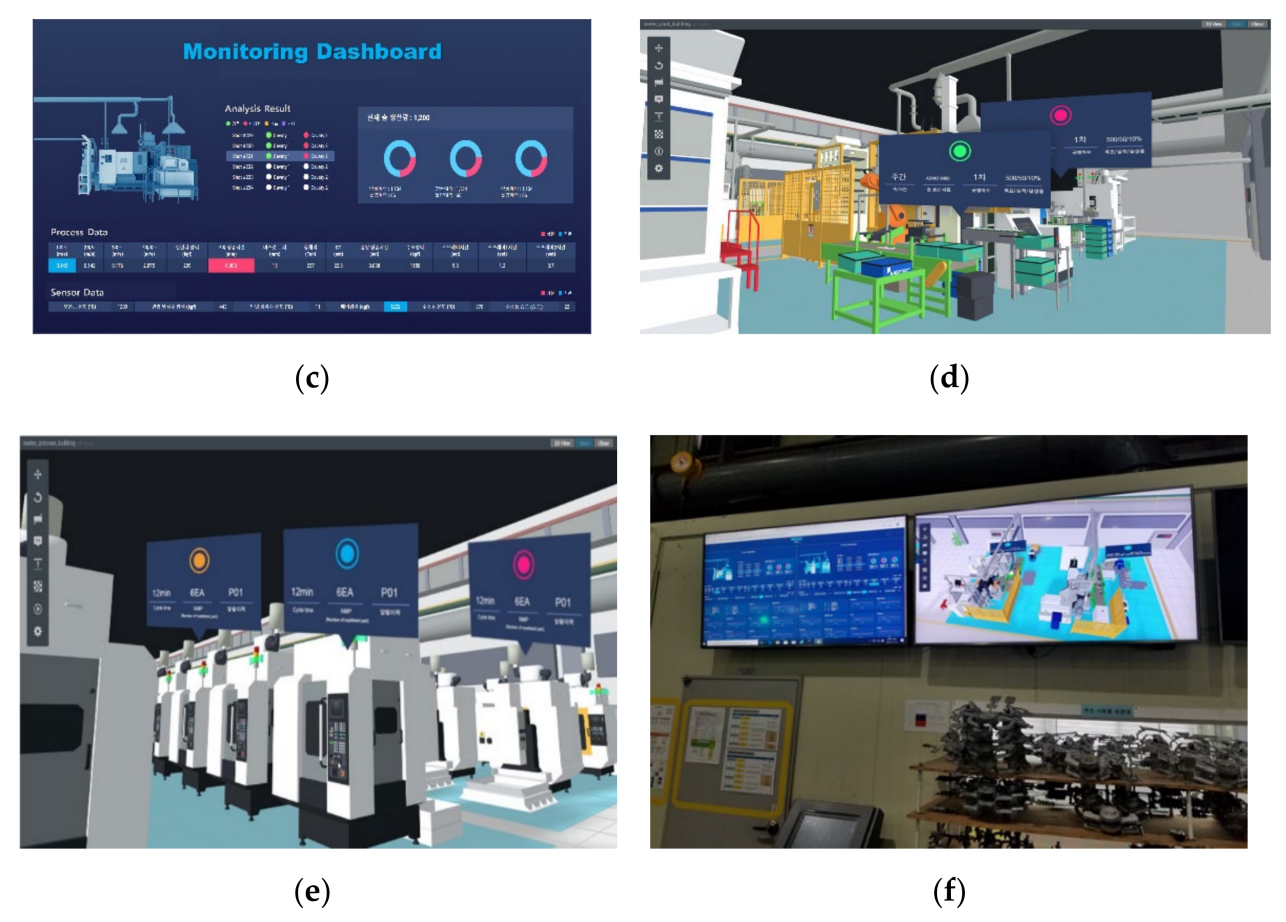
Application Case of the company “N”: (**a**) Die-Casting Machines, (**b**) CNC Machines, (**c**) 2D Chart Dashboard, (**d**) 3D Dashboard of Die-Casting Machines, (**e**) 3D Dashboard of CNC Machines, (**f**) Screenshot of Shop floor.

**Figure 6:**
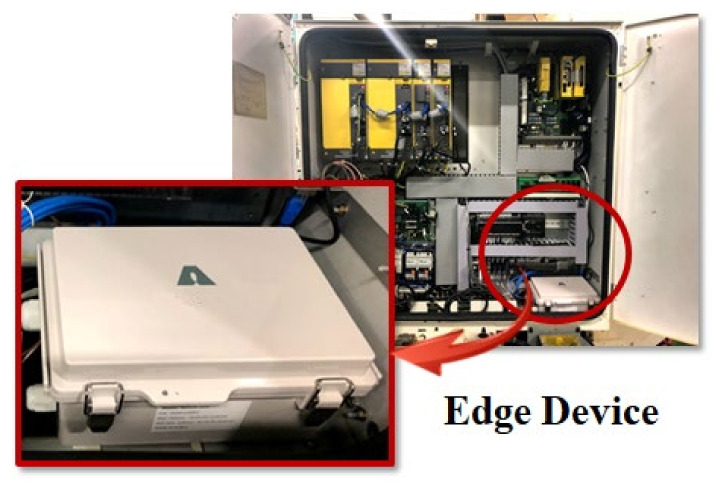
Installed edge devices.

Figure 5 shows the machines of the company’s manufacturing site and the implemented digital twin-based integrated monitoring system. Figure 5a is the die-casting machines of the target factory, and (b) is the CNC machines. Figure 6 shows the edge device installed in the site. Data and KPIs are being collected from DB of legacy system including real-time data collected from edge devices and MES used by the company. Operation status, production products, purpose, performance, achievement rate, cycle time, alarm history, real-time sensing data such as temperature, pressure, humidity, and failure prediction results are collected from die-casting and CNC machines, and it is being visualized along with a realistic 3D digital twin model.

Figure 5c is a 2D chart-based dashboard showing the KPIs and data collected in the casting machine. Production data, product name, mold number, speed, high speed, cylinder pressure, high speed rise time, casting pressure, spray time, etc., are visualized in real time. In connection with the data analysis module, abnormal situations are automatically detected. Figure 5d shows the appearance of the digital twins of the casting machines. When it is in normal operation, the machine is moving and working, but when it is not in operation, the machine also stops working and the user can intuitively check the status through an alarm. Figure 5e is the digital twins of the CNC machines. The operation status of the machine can be intuitively identified, and an alarm is generated when an abnormal activity occurs in the machine. Figure 5f is the appearance of the monitoring dashboard installed at the worksite. This allows site workers to intuitively learn and grasp the current machines and factory conditions. 

**Figure 7:**
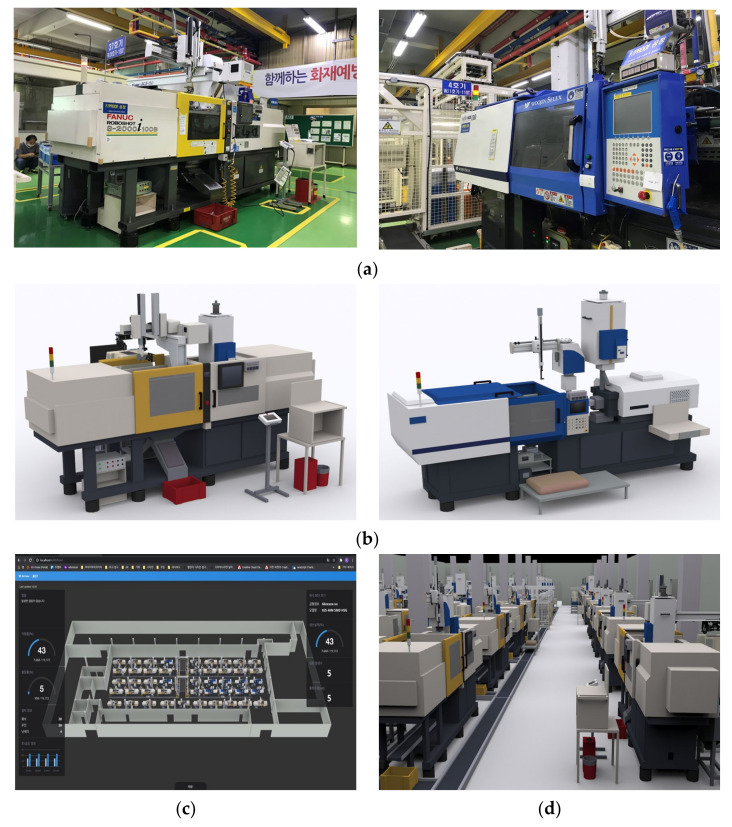
Application Case of the company “K”: (**a**) Injection Molding Machines, (**b**) 3D model of Injection Molding Machines, (**c**) Integrated Dashboard of Shopfloor, (**d**) Work-through mode.

The company “K” supplies modules for eco-friendly and intelligent automobiles, and connectors for digital home appliances and IT devices. It is a global manufacturing company that has their headquarters in South Korea and operates 13 factories in Asia, Europe, and America. The company “K” also used the edge device as shown in Figure 6 and built a digital twin-based control and monitoring system in the same way as company “N”. Figure 7a shows the target injection molding machines. Figure 7b shows the 3D modelled machines, and Figure 7c shows the entire digital twin of the shopfloor. On the left and right sides, information on temperature and humidity of the factory, operation status, machines, and molds are provided in the form of charts.

Earlier, it was mentioned that manufacturing companies must meet three criteria to introduce a digital twin-based monitoring system using realistic 3D models. The first was that users of manufacturing companies should be able to reflect frequently changing manufacturing environments in the digital twin. Engineers of applied companies are easily handling layout change tasks using the layout design tool used in this study. Users in manufacturing companies have learned how to use the system through training within an hour, and they are quickly and easily creating digital twins in the desired form. 

The second criterion was that 3D should be conveniently or automatically generated, and 3D visualization should support all cloud, web, and mobile. The target factories of the applied companies consist of a casting factory, an inspection factory, a CNC machining factory, injection molding factory, and a warehouse. Three-dimensional factories are automatically created according to the layout changed in the authoring tool. The developed monitoring system visualizes the digital twin of the entire factory on mobile, web, and cloud, and operates well without any performance problems. In addition, the comparison of 3D files is summarized in Table 2. It was confirmed that the file size of the 3D library was reduced to within 10% of the original CAD. This is a comparison for one machine, and more diverse cases are needed for an objective comparison.

Third, it is necessary to be connected to the site in real time to visualize data and KPIs along with 3D models and provide user-friendly functions. Figure 5 shows the real-time visualization of the 3D models and KPIs. There is an autorotation function within the digital twin, which provides automatic rotation of the entire factory to the user. It supports a function that allows users to display screens when they set points that they want to check specifically (e.g., specific machine or material handling system). It is also possible to measure distance of objects in the digital twin, and various convenient functions such as the walkthrough are provided. The data collected in real time are connected to the analysis module to provide abnormal situation detection and alarm generation functions. 

Previously, in order to check the operation status and KPIs of the factory at the production site, it was necessary to look at the machines directly on the shop floor and then go to the office to check the factory KPIs and the status of each machine through MES. However, through the establishment of a digital twin monitoring system, the situation of the site can be immediately checked anytime, anywhere. There was feedback from customers that the speed of handling work and decision-making time increased by more than 30% compared to the previous one.

## 5. Conclusions

In this study, in order to increase the understanding of digital twin building of SMEs, the tasks and technologies for each layer were separately explained based on a single digital twin architecture. In addition, by developing a digital twin solution for SMEs, the technology and tasks of each layer were integrated. The creation of digital twin models, which SMEs struggle most with, has been automated through system development. A 3D digital twin can be created automatically after reflecting the frequently changing worksite conditions by the manufacturing company users themselves. By connecting to the site in real time based on the edge devices, it is possible to intuitively check the current factory status with KPIs of a realistic 3D-based digital twin through cloud, web, and mobile. Various user-friendly functions such as automatic camera rotation and movement, measurement, walk-through, and VR functions are provided.

Beyond simply viewing the production site with CCTV, it has become possible to monitor and manage production sites based on digital twins. The factory can be intuitively inspected anytime, anywhere, and the data analysis function has been established, enabling it to identify problems and make quick decisions. The big advantage of digital twins is that users can interact according to circumstances based on realistic 3D. The user can select the machines or material handling systems in which the problem has occurred through the interaction to determine the cause of the malfunction in detail, select and execute the necessary analysis algorithm, and even control. Currently, manufacturing companies want minimal human intervention to prevent accidents that may occur due to automatic control. 

The digital twin-based integrated monitoring system can be connected to various MES and IoT platforms based on the RESTful API and can be integrated with heterogeneous analysis systems. Currently, monitoring systems are being built in connection with various legacy environments in various manufacturing industries. Currently, the asset administration shell (AAS) [37] is being applied to the digital twin systems by integrating with the interoperable data model. AAS is the implementation of the digital twin for Industry 4.0 and can support interoperability between systems based on digital twins [38]. Therefore, we intend to implement it to support interoperability between various systems by connecting elements of the proposed FDI data model and properties of the AAS meta model. There is a plan to perform a quantitative evaluation of the system based on the accumulated application cases in the future.

## Figures and Tables

**Table 1:** Element of FDI data model.

Element	Description
Product	Product type, parts that make up the product
Machine	Sensing information, setting value (setup time, cycle time, MTTR, MTBF, etc.)
Labor	Gender, working hours, skill, etc.
Material Handling System	Material handling module ID (Ex: Container), quantity, cycle time, etc.
Material Handling Module	Part ID, quantity, etc.
Routing	Process order and distance, etc.
Process	Standby, loading, operation, unloading, setup, information, etc.
Layout	Building, floor, geometry information, process area information, etc.
KPI	Respond ability, OEE, automation rare, space utilization, yield, throughput, energy consumption, CO2 emission, etc.
Rule	Legal aisle width, number of gaps between columns, legal regulations such as door position, etc.
SimulationModelInfo	Information related to the model by the simulation (e.g., simulation purpose, tool information etc.)
SimulationRunInfo	Information related to the simulation performance (e.g., simulation execution time, etc.)
UnitofMeasurement	Units used in the schema (e.g., length in m, time in sec, etc.)

**Table 2:** Comparison of file format.

Type	Main Data	Size
Original CAD	History, Constraints, PMI, BREP, Attributes, Facets	
Lightweight file	PMI, Precise BREP, Attributes, LODs, Bounding Box	<30%
Proposed file	LOD, Tessellation, Attributes, Texture, Animation	<10%

## Data Availability

Not applicable.
